# Getting the food list ‘right’: an approach for the development of nutrition-relevant food lists for household consumption and expenditure surveys

**DOI:** 10.1017/S1368980018002847

**Published:** 2018-11-05

**Authors:** Winnie Bell, Jennifer C Coates, Beatrice L Rogers, Odilia I Bermudez

**Affiliations:** 1 Tufts University Friedman School of Nutrition Science and Policy, 150 Harrison Avenue, Boston, MA 02111, USA; 2 Tufts University School of Medicine, Boston, MA, USA

**Keywords:** Food list development, Household consumption surveys, Dietary data, Food consumption data, Bangladesh

## Abstract

**Objective:**

The present paper aimed to demonstrate how 24 h dietary recall data can be used to generate a nutrition-relevant food list for household consumption and expenditure surveys (HCES) using contribution analysis and stepwise regression.

**Design:**

The analysis used data from the 2011/12 Bangladesh Integrated Household Survey (BIHS), which is nationally representative of rural Bangladesh. A total of 325 primary sampling units (PSU=village) were surveyed through a two-stage stratified sampling approach. The household food consumption module used for the analysis consisted of a 24 h open dietary recall in which the female member in charge of preparing and serving food was asked about foods and quantities consumed by the whole household.

**Setting:**

Rural Bangladesh.

**Participants:**

A total of 6500 households.

**Results:**

The original 24 h open dietary recall data in the BIHS were comprised of 288 individual foods that were grouped into ninety-four similar food groups. Contribution analysis and stepwise regression were based on nutrients of public health interest in Bangladesh (energy, protein, fat, Fe, Zn, vitamin A). These steps revealed that a list of fifty-nine food items captures approximately 90 % of the total intake and up to 90 % of the between-person variation for the key nutrients based on the diets of the population.

**Conclusions:**

The study illustrates how 24 h open dietary recall data can be used to generate a country-specific nutrition-relevant food list that could be integrated into an HCES consumption module to enable more accurate and comprehensive household-level food and nutrient analyses.

Many low- and middle-income countries face a dual burden of malnutrition, with high rates of undernutrition alongside overweight and obesity^(^ ^1^ ^)^. Due to under-investment in individual-level dietary data in many low- and middle-income countries, detailed knowledge about dietary patterns is largely lacking. In an effort to fill this gap, researchers have turned to alternative data sources, including household consumption and expenditure surveys (HCES). Traditionally, the primary uses of HCES have been to measure and monitor poverty, as a basis for calculating consumer price indices, and assembling national accounts^(^ ^2^ ^)^. HCES are also known as household budget surveys (HBS), household income and expenditure surveys (HIES) and living standard measurement surveys (LSMS), and are conducted on a semi-regular basis in most low-, middle- and high-income countries^(^ ^2^ ^)^.

Consumption modules in HCES vary greatly from country to country. Some of the key ways in which the consumption modules differ include: (i) the length of the recall period; (ii) whether data are collected for acquisition, consumption or both; (iii) whether there is information on the mode of food acquisition (purchases, own production and in-kind); (iv) whether or not information on food consumed away from home is collected and in what form; and (v) whether food details are collected through open recall or a list, and in what form^(^ ^2^ ^–^ ^4^ ^)^. The latter issue is the focus of the present paper.

More specifically, consumption data in HCES can be collected using several different methods, including open recall, diary method or food list-based recall, in order to capture the household expenditure or actual food consumption. Not all HCES include a food list in their consumption modules, but using a food list-based approach is advantageous for several reasons. For example, in comparison to open recalls and diary methods, food list-based modules could be less costly and time-consuming to implement once the food list has been created. Furthermore, a food list requires limited training of the enumerator compared with open recall methods and could result in more consistent results as enumerators are guided by the food list. In addition, relying on a food list is less burdensome to respondents and can work well with people of varied literacy compared with diary methods (which typically require that the respondent be literate). However, if a food list is used, the composition and length of the food list are important, as the comprehensiveness of the data hinges on the quality of the food list.

One benefit of developing a comprehensive food list is that it allows for data from the HCES to be used for food and nutrition purposes, in addition to the common uses of poverty measurement, calculating consumer price indices and assembling national accounts. Furthermore, from a food security and nutrition perspective, a comprehensive HCES food list can also be used to calculate household-level food and nutrient availability and dietary diversity, identify potential food fortificant vehicles, and estimate food expenditure (or value of food consumption) as a proportion of total household expenditure. These dietary and food security indicators are additional to the types of indicators for which HCES are typically designed. While there is likely substantial overlap among foods included in the list developed for each of these purposes, the level of specificity required of food items is likely greatest for conducting analyses related to nutrient consumption because all foods need to be matched to their nutrient profiles in the food composition table (FCT).

The field of nutritional epidemiology has well-established methods for developing FFQ, which are essentially food lists designed to capture estimated usual intake of key foods or nutrients based on consumption over a multi-day reference period^(^ ^5^ ^–^ ^7^ ^)^. An FFQ that is developed ‘from the ground up’ generally starts with a recent quantitative 24 h dietary recall or weighed food record data from a representative sample of the population in which the questionnaire will be administered^(^ ^8^ ^,^ ^9^ ^)^. The traditional approach, recommended by Block *et al.* ^(^ ^8^ ^)^, is to rank food items by the percentage each food contributes to the total intake of a given nutrient and then use a chosen cut-off to select foods. The development of an FFQ ideally takes into account both the total intake of foods and the extent to which consumption of specific foods differs between individuals (i.e. between-person variation) by considering the frequency of consumption, the proportion of people consuming the food and the overall contribution of a given food to the intake of the nutrient(s) or food group(s) of interest^(^ ^9^ ^)^. The methods used to develop an FFQ provide a potentially useful model for developing a comprehensive food list for HCES consumption modules.

As with HCES, the level of disaggregation as well as the length of the food list affect the accuracy of the data. In a review of 223 FFQ, Cade *et al.* ^(^ ^9^ ^)^ found that the number of foods ranged from five to 350, with the average being seventy-nine items. Earlier research comparing an FFQ with a food diary during a controlled feeding study in the USA found that individual food names elicit more accurate recall responses than generic names (e.g. ‘orange’ *v*. ‘fruit’)^(^ ^10^ ^)^. However, FFQ range in length in part because they are developed for different purposes. For example, only eleven items were required for an FFQ designed to measure Ca and vitamin D intakes in a senior population in Scotland^(^ ^6^ ^)^, compared with a food list that attempts to capture all foods consumed. Food lists also vary in length because they are tailored to the characteristics of the study population. For example, an FFQ for use in a population with multiple ethnicities with diverse diets would require a longer food list than an FFQ for a single ethnic group that consumes a homogeneous diet^(^ ^11^ ^)^. Generally, nutritional epidemiological studies show that there are diminishing returns to longer food lists due to time, cost and respondent fatigue.

The present paper contributes to the growing methodological literature regarding deriving food consumption information from HCES in order to provide recommendations to HCES survey developers and end users of these data. This work is part of a larger effort being carried out by the International Dietary Data Expansion (INDDEX) Project to increase the availability, accessibility and use of individual and household food consumption data^(^ ^12^ ^)^. Other papers with the same overarching purpose have addressed issues related to collecting data on food away from home, nutrient imputation, the number of meal partakers and validation of the Adult Male Equivalent method for estimating the adequacy of nutrient intakes from household data^(^ ^13^ ^–^ ^15^ ^)^. The specific objective of the present paper is to demonstrate how 24 h dietary recall data can be used to generate a food list for the consumption module in HCES that can capture intakes of key nutrients with a closed FFQ-style survey. Using dietary data from the large-scale Bangladesh Integrated Household Survey (BIHS), the paper assesses the composition, length and level of disaggregation of the food list necessary to derive accurate energy and nutrient intake estimates at the household level.

## Methods

### Data source

The BIHS is a three-round panel survey conducted by the International Food Policy Research Institute with support from the US Agency for International Development. The present study relied on the first round of the survey conducted in 2011/12 and released in 2013. The data for this round were collected between October 2011 and March 2012, spanning the late autumn (*hemanta*) and winter (*shit*) in Bangladesh. A second round of data was collected in 2015 and released at the end of 2016 (when the current analysis was already underway).

The BIHS is a unique data set that contains information including plot-level agricultural production and practices, household dietary intake and allocation to individual household members, and anthropometric measurements of all household members^(^ ^16^ ^)^. The household food consumption module was used for the current analysis and consisted of a 24 h open dietary recall in which the female member in charge of preparing and serving food was interviewed and asked about foods and quantities consumed by the whole household. The total number of foods (individual items and composite dishes) reported in the 24 h open dietary recall was 288 individual items.* Ingredients were reported for each household recipe or composite dish (i.e. standardized recipes were not used in the first round of the survey in 2011/12) and food consumed away from home was reported as a composite dish without details on the dishes’ ingredients. The data are nationally representative of rural Bangladesh and of rural areas of seven administrative divisions (Barisal, Chittagong, Dhaka, Khulna, Rajshani, Rangpur, Sylhet), as well as representative of the Feed the Future zone of influence. A total of 6500 households in 325 primary sampling units (PSU=village) were surveyed through a two-stage stratified sampling approach.

### Development of nutrient analytic file

To understand the contributions of individual food items and groups of foods to households’ nutrient intakes, the first step was to match the reported foods with corresponding food items in the *Food Composition Table for Bangladesh* developed by the Institute of Nutrition and Food Science Centre for Advanced Research in Sciences at the University of Dhaka^(^ ^17^ ^)^. Individual and composite foods that were not identified in the Bangladesh FCT were matched with foods from the US Department of Agriculture’s Food Composition Database (USDA FCDB) for Standard Reference, release 22^(^ ^18^ ^)^. One of the authors previously used the USDA FCDB in research with the Bangladesh Household Income and Expenditure Survey (BHIES) and had already recreated the nutrient values for some of the composite and processed foods^(^ ^19^ ^)^. Based on this approach, 81 % of food matches were made using the Bangladesh FCT and the remaining 19 % of matches were made with the USDA FCDB.

The following nutrients of public health interest were selected for the present study: energy, fat, protein, Fe, Zn and vitamin A^(^ ^1^ ^)^.† By convention, values of per capita daily energy consumption <2092kJ (<500 kcal) and >20 920 kJ (>5000 kcal) are considered extreme values and were eliminated^(^ ^19^ ^,^ ^20^ ^)^. This approach resulted in a final sample size of 6374 households.

### Analytical approach

An ideal food list must capture the variability of the population’s diet, captured both in absolute and in relative terms. To accomplish this, a recently developed four-step process^(^ ^11^ ^,^ ^21^ ^)^ was followed that builds on a classic methodology developed by Block *et al*. to develop a food list based on the predetermined set of nutrients of interest^(^ ^8^ ^)^. Each step is described below, sequentially, keeping in mind that the objective of this overall process was to identify a food list that captures the key foods in the diet that span the nutrients of interest, while maintaining as many distinct foods with one-to-one matches in the FCT as possible.

The goal of the first step was to aggregate the individual foods consumed by less than 2 % of the population or in a few cases foods that shared the same underlying values in the FCT. The individual identity of foods was maintained if 2 % or more of the population consumed them, with the objective of ensuring as many direct matches in the FCT as possible. Approximately 73 % of all foods (211 of 288 foods) were consumed by less than 2 % of the population and therefore we determined that these foods should be maintained by aggregation rather than removing them from the food list entirely.

Foods consumed by less than 2 % of the population were grouped together based on comparable nutrient composition – specifically, energy and fat for staples and meats, and vitamins and minerals of interest for fruits and vegetables – or put into an ‘other’ category (e.g. ‘fruits, other’) following examples from the literature^(^ ^11^ ^,^ ^21^ ^–^ ^23^ ^)^. For example, many fruits were consumed by less than 2 % of the population, so several different subgroupings were made for similar categories of fruit. Some examples of fruit subgroups include: (i) citrus (e.g. orange, pomelo), (ii) energy-rich fruits (e.g. dates, tamarind) and (iii) other fruits (e.g. apples, grapes); while any fruits consumed by 2 % or more of the population (e.g. bananas, coconuts) were left as individual items. A few exceptions to this rule were made independent of the percentage consuming in cases where foods were consumed by a large proportion of the population but the underlying FCT values were the same due to limited composition data. For example, parboiled coarse rice and non-parboiled coarse rice were matched to the same underlying value for coarse rice and were therefore grouped together.

Ultimately, this aggregation process resulted in a total of ninety-four minimally aggregated foods (i.e. groupings of those individual foods consumed by <2 % of the population), effectively reducing the food list from 288 foods to ninety-four minimally grouped foods. While all composite dishes consumed in the home reported by households were collected as individual foods, composite dishes consumed away from home were reported as a dish. Therefore, composite dishes consumed away from home were matched with previously constructed FCT recipe data. Most of these were consumed by less than 2 % of the respondents and thus were aggregated as explained above; however, some composite dishes and prepared foods were consumed by 2 % or more of the population (e.g. *bonroti*/*paoroti*) and in these cases the items were left as single foods for the analysis.

The objective of the second step was to rank the ninety-four minimally grouped foods based on their contribution to total population intake of a given nutrient. The contribution analysis and ranking of foods were carried out for the food items once they were grouped (*n* 94). This step is referred to as ‘contribution analysis’ in nutritional epidemiology and follows the method detailed by Block *et al.* ^(^ ^8^ ^)^. Similar to Kobayashi *et al*.^(^ ^21^ ^)^, the ranking was based on the percentage of priority nutrient that each food contributed to the total reported nutrient intake of all survey respondents. For example, in the case of vitamin A, the total amount of vitamin A consumed was divided by the vitamin A contributed by each individual food to determine the percentage contribution. The ranking for the ninety-four foods was used to determine the final food list. Any foods that contributed cumulatively to at least 90 % of total intake of the selected nutrient were retained for the final food list.

The purpose of the third step was to identify the foods with the highest between-person variance for the intake of the pre-specified nutrients using a forward stepwise regression. While the contribution analysis (the second step described above) is the traditional way of selecting foods for an FFQ, this additional step has emerged in the literature as a complementary way of determining important foods in the diet based on the variation in consumption^(^ ^11^ ^,^ ^21^ ^–^ ^23^ ^)^. The stepwise regression is important to identify foods that, on average, may not be major contributors to total intake but provide a substantial amount of a given nutrient for a specific subgroup of the population.

Using a stepwise regression with forward selection and a relaxed entry criterion of *P*<0·6, we first ranked the independent variables in descending order of extra variance of the dependent variable explained (e.g. total energy, total vitamin A). Then, following the same order, we manually added the independent variables (e.g. energy per food item, vitamin A per food item) sequentially into the model until the cumulative *R* ^2^ of the model exceeded 0·90. The ninety-four foods were used for the forward stepwise regression; the dependent variable in the model was the total amount of each nutrient or energy consumed and the independent variables entered sequentially were the quantity of that nutrient or energy consumed for each given food item. Independent variables (each additional food) were added to the model until at least 90 % of the variability of that nutrient or energy was explained by the independent variables based on the coefficient of determination (i.e. the cumulative *R* ^2^).

The fourth and final step was to reconcile the food lists generated in the second step of the contribution analysis with the foods identified in the third step from the forward stepwise regression. This approach followed previous examples from the literature^(^ ^8^ ^,^ ^11^ ^,^ ^21^ ^)^. Thus, the final food list includes foods that contributed cumulatively at least 90 % of the intake of any of the nutrients analysed as well as those that contributed to accounting for at least 90 % of between-person variability for the preselected list of nutrients. Microsoft^®^ Excel version 15.38 was used for the preliminary food matching and creation of the nutrient analytic file. These items were then matched with the food items from the BIHS in the Stata statistical software package. All analyses were conducted using Stata version 14.

## Results

### Total intake and contribution analysis

**Fig. 1:**
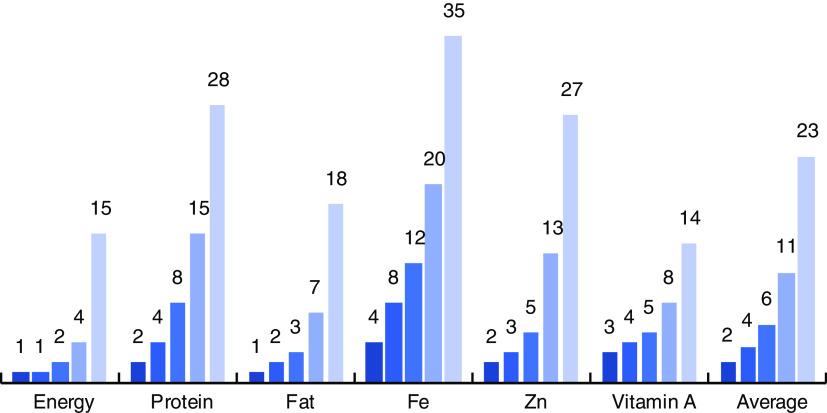
(colour online) Number of foods (out of a total of ninety-four) required to meet different levels of total intake (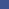, 50 %; 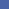, 60 %; 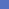, 70 %; 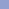, 80 %; 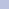, 90 %), by nutrient, among 6374 Bangladeshi households using data from the 2011/12 Bangladesh Integrated Household Survey (BIHS)

Of the ninety-four foods, on average, only two foods were needed to meet 50 % of total intake for energy, protein, fat, Fe, Zn and vitamin A, while twenty-three of the ninety-four foods were needed to account for 90 % of reported intake for the same nutrients. The total number of foods required to meet 90 % of nutrient intake ranged from a low of fourteen foods for vitamin A to a high of thirty-five foods for Fe (Fig. 1).

The remainder of the results focus on energy and vitamin A to illustrate the process and findings. Table 1 shows the top twenty contributors, among the ninety-four individual foods, according to their percentage contribution to total energy intake. Over 70 % of energy in the rural Bangladeshi diet comes from two different types of rice: coarse rice, which includes parboiled and non-parboiled rice; and fine rice. The top food contributors to total energy intake were commonly consumed by a large proportion of the population. For example, the different types of rice, soyabean oil, wheat flour and potatoes together account for nearly 83 % of total energy intake, and the coarse rice, soyabean oil and potatoes are each consumed at least once per day by more than 80 % of the households.

**Table 1:** Top twenty food contributors to total dietary energy intake (out of a total of ninety-four foods) among 6374 rural Bangladeshi households using data from the 2011/12 Bangladesh Integrated Household Survey (BIHS)

		Contribution to total intake		
Rank	Food item	Percentage	Cumulative percentage	Households consuming specific food (%)
1.	Rice, coarse	63·47	63·47	89·52
2.	Rice, fine	7·52	71·00	12·96
3.	Soyabean oil	6·30	77·30	83·02
4.	*Atta* (wheat flour)	2·89	80·19	15·09
5.	Potato	2·51	82·70	80·88
6.	Rice flour	1·26	83·96	5·74
7.	Mustard oil	1·17	85·13	31·47
8.	*Muri*/*khoi* (puffed rice)	1·15	86·28	16·88
9.	Sugar	0·79	87·07	35·28
10.	Lentil	0·71	87·78	13·32
11.	Biscuit	0·60	88·38	26·81
12.	Large fish, other	0·55	88·93	16·57
13.	Onion	0·53	89·46	95·95
14.	Dried fish	0·44	89·90	14·34
15.	Sweeteners, other	0·38	90·28	6·48
16.	Coconut	0·37	90·65	2·76
17.	Beef/buffalo	0·36	91·01	4·77
18.	Milk	0·35	91·36	18·15
19.	Dried chilli	0·34	91·70	69·19
20.	Small fish, other	0·33	92·03	11·08

Foods contributing to total vitamin A intake show a different pattern (Table 2). The top twenty sources of vitamin A include various types of leafy, green vegetables and dried chilli. Leafy greens are a key source for vitamin A intake in Bangladesh, with amaranth leaves and other leafy vegetables contributing nearly 50 % to total vitamin A consumption. Results for protein, fat, Fe and Zn can be found in the online supplementary material, Supplemental Tables 1 to 4, respectively.

**Table 2:** Top twenty food contributors to total vitamin A (retinol activity equivalents) intake (out of a total of ninety-four foods) among 6374 rural Bangladeshi households using data from the 2011/12 Bangladesh Integrated Household Survey (BIHS)

		Contribution to total intake		
Rank	Food item	Percentage	Cumulative percentage	Households consuming specific food (%)
1.	Amaranth leaves	23·81	23·81	6·92
2.	Leafy vegetables, other	23·52	47·32	9·81
3.	Dried chilli	10·01	57·33	69·19
4.	Spinach	8·19	65·53	4·83
5.	Large fish, other	4·85	70·38	16·57
6.	Egg	4·39	74·77	15·42
7.	*Lau shak* (bottle gourd leaves)	4·33	79·10	5·19
8.	*Dhania shak* (leafy vegetable)	2·65	81·75	17·90
9.	*Sheem* (bean)	2·38	84·13	27·57
10.	Offal	1·58	85·71	0·47
11.	Milk	1·44	87·15	18·15
12.	Taki fish	1·16	88·30	1·66
13.	Puti fish	1·05	89·35	6·06
14.	Potato	1·05	90·40	80·88
15.	Koi fish	0·95	91·35	2·04
16.	Chicken	0·77	92·12	6·31
17.	Small fish, other	0·77	92·89	11·08
18.	Tomato	0·67	93·56	18·45
19.	Eggplant	0·62	94·18	31·60
20.	Water gourd	0·59	94·77	7·70

### Stepwise regression and between-person variance

**Fig. 2:**
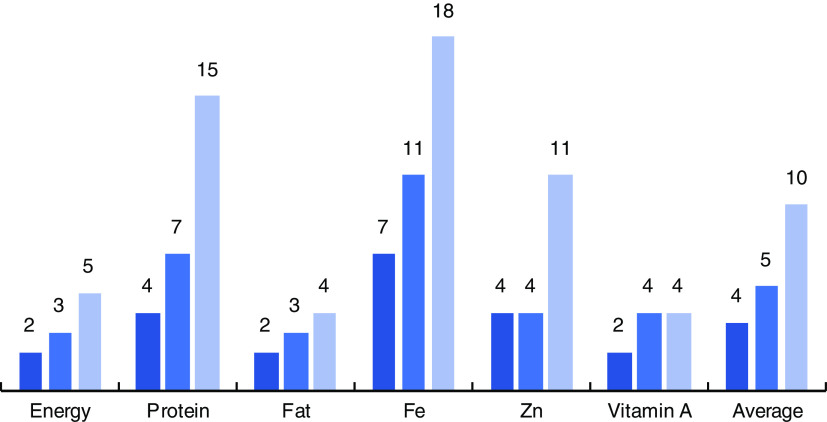
(colour online) Number of foods (out of a total of ninety-four) required to explain different levels of between-person variation (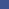,70 %; 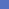, 80 %; 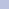, 90 %), by nutrient, among 6374 rural Bangladeshi households using data from the 2011/12 Bangladesh Integrated Household Survey (BIHS)

In contrast to the total number of foods required in the contribution analysis, even fewer foods were required to explain the between-person variation for the same nutrient components. On average four, five and ten foods explained 70, 80 and 90 % of the between-person variation, respectively, for energy, protein, fat, Fe, Zn and vitamin A (Fig. 2). The smallest number of foods required to explain 90 % of the between-person variation was for energy, fat and vitamin A (five, four and four foods, respectively). On the other hand, explaining 90 % of the between-person variation for Fe required eighteen foods, protein required fifteen foods and Zn required eleven foods (see online supplementary material, Supplemental Tables 5 to 8, for specific food items).

Table 3 distinguishes the foods required to explain 90 % of between-person variation as different from those required for total intake. Information on between-person variation provides details on the foods that provide an important source of variation to the total nutrient intake, often consumed by a subgroup of the population, while the total intake simply ranks the most consumed foods based on contribution of nutrient intake to total. The items in Table 3 are organized from highest to lowest between-person variation from each additional food (as measured by the coefficient of determination (*R* ^2^)), which corresponds to the order in which items were entered into the stepwise regression (as shown in the first column). The first fifteen foods explain about 98 % (*R* ^2^=0·978) of between-person variation of energy, with three different forms of rice explaining over 80 % (coarse rice, fine rice, rice flour). However, when looking at the contribution to total energy intake, these same fifteen foods – ordered according to degree of explanation of between-person variation, not total intake – account for 87 % of total energy. In comparison, the first fifteen foods in Table 1 contribute 92 % of total energy intake, further highlighting the difference between the contribution analysis and between-person variation.

**Table 3:** Contribution to between-person variation and to total dietary energy intake for the top fifteen foods (out of a total of ninety-four) accounting for variance in energy intake among 6374 rural Bangladeshi households using data from the 2011/12 Bangladesh Integrated Household Survey (BIHS)

			Contribution to between-person variation of energy	Contribution to total energy intake		
Stage of entry into regression*	Food item	Households consuming specific food (%)	Cumulative *R* ^2^	Rank for total intake†	Percentage	Cumulative percentage
1.	Rice, coarse	89·52	0·470	1	63·47	63·47
2.	Rice, fine	12·96	0·782	2	7·52	70·99
3.	Rice flour	5·74	0·832	6	1·26	72·25
4.	*Atta* (wheat flour)	15·09	0·881	4	2·89	75·14
5.	Soyabean oil	83·02	0·923	3	6·30	81·44
6.	*Muri*/*khoi* (puffed rice)	16·88	0·937	8	1·15	82·59
7.	Mustard oil	31·47	0·946	7	1·17	83·76
8.	Sugar	35·28	0·955	9	0·79	84·55
9.	Coconut	2·76	0·960	16	0·37	84·92
10.	Grains, other	3·48	0·964	27	0·28	85·20
11.	*Maida* (wheat flour w/out bran)	3·03	0·968	26	0·28	85·48
12.	Biscuit	26·81	0·971	11	0·60	86·08
13.	Prepared foods	2·38	0·974	37	0·19	86·27
14.	Sweeteners, other	6·48	0·976	15	0·38	86·65
15.	Beef/buffalo	4·77	0·978	17	0·36	87·01

* Stage of entry based on contribution to variance in consumption, ranked high to low.

† Ranked in order of highest to lowest contributor to total nutrient.

More specifically, for example, rice flour entered into the stepwise regression third, yet is ranked sixth for total intake. More strikingly, prepared foods, which were ranked thirty-seventh for total intake, were entered thirteenth into the stepwise regression for contribution to between-person energy intake. The stepwise regression determined that five of the top fifteen foods from the contribution analysis (Table 1) were not important for explaining between-person variation: potatoes, lentils, onions, other large fish and dried fish. While these are in the top fifteen foods that contribute to 90 % of total intake according to the contribution analysis, when it comes to the stepwise regression they are not included because they do not provide additional explanation of the variation in intake after accounting for the other items already included in the regression. This demonstrates that these two approaches are complementary, and food items from the two approaches must be reconciled to arrive at the final food list.

Similarly, Table 4 shows the contribution of different foods to vitamin A intake. In this case four foods – amaranth leaves, leafy vegetables, spinach and organ meat – explain over 90 % of total between-person variation, while the top fifteen foods explain nearly 100 % of between-person variation. While these fifteen foods explain virtually all of the between-person variation (*R* ^2^=0·996), these same foods explain only about 90 % of total vitamin A intake. Items such as offal (organ meat), which was selected for entry into the regression fourth due to its large coefficient of determination (*R* ^2^), was ranked tenth when considering the contribution to total intake of vitamin A. On the other hand, in the contribution analysis (Table 1), the items ranked third (dried chilli) and fourteenth (potatoes) were excluded from Table 4 due to the ranking for the stepwise regression.

**Table 4:** Contribution to between-person variation and to total vitamin A (retinol activity equivalents) intake for the top fifteen foods (out of a total of ninety-four) accounting for variance in vitamin A intake among 6374 rural Bangladeshi households using data from the 2011/12 Bangladesh Integrated Household Survey (BIHS)

			Contribution to between-person variation of vitamin A	Contribution to total vitamin A intake		
Stage of entry into regression*	Food item	Households consuming specific food (%)	Cumulative *R* ^2^	Rank for total intake†	Percentage	Cumulative percentage
1.	Amaranth leaves	6·92	0·476	1	23·81	23·81
2.	Leafy vegetables, other	9·81	0·719	2	23·52	47·33
3.	Spinach	4·83	0·784	4	8·19	55·52
4.	Offal	0·47	0·914	10	1·58	57·10
5.	Large fish, other	16·57	0·949	5	4·85	61·95
6.	*Lau shak* (bottle gourd leaves)	5·19	0·964	7	4·33	66·28
7.	Dried chilli	69·19	0·975	3	10·01	76·29
8.	Egg	15·42	0·982	6	4·39	80·68
9.	*Dhania shak* (leafy vegetable)	17·90	0·987	8	2·65	83·33
10.	Taki fish	1·66	0·990	12	1·16	84·49
11.	Vegetables (vitamin A rich), other	1·24	0·992	21	0·52	85·01
12.	Koi fish	2·04	0·994	15	0·95	85·96
13.	Milk	18·15	0·995	11	1·44	87·40
14.	*Sheem* (bean)	27·57	0·996	9	2·38	89·78
15.	Puti fish	6·06	0·996	13	1·05	90·83

* Stage of entry based on contribution to variance in consumption, ranked high to low.

† Ranked in order of highest to lowest contributor to total nutrient.

In rural Bangladesh fewer foods are needed to explain between-person variance in the diet than the (slightly) larger number of foods required to meet similar levels of total intake. In addition, the number of foods that explain different levels of between-person variation, as well as total intake, vary by nutrient.

### Final food list

To create the final food list, the results from the contribution analyses and stepwise regression were combined. Foods that either contributed cumulatively to 90 % of key nutrient intake or explained up to 90 % of between-person variability were included in the final food list (Table 5). This approach followed previous examples from the literature^(^ ^8^ ^,^ ^11^ ^,^ ^21^ ^)^. The food list was developed based on comparative analysis of all items across the different analyses for the predefined nutrients of interest: energy, protein, fat, Fe, Zn and vitamin A. After comparing and compiling foods, the final food list contained fifty-nine items, classified into nine food groups. Table 5 shows the final food items and the food groups.

**Table 5:** Comprehensive food list for rural Bangladesh (*n*=59)

Food Category: Grains		Food Category: Vegetables (*continued*)		Food Category: Meat		Food Category: Eggs & Dairy	
1.	Rice, coarse	15.	Eggplant	30.	Beef/buffalo	46.	Egg
2.	Rice, fine	16.	Garlic	31.	Chicken	47.	Milk
3.	Rice flour	17.	Green chilli	32.	Offal		
4.	*Muri*/*khoi* (puffed rice)	18.	*Lau shak* (bottle gourd leaves)	33.	Birds, other	Food Category: Spices	
5.	*Atta* (Wheat flour)	19.	Onion	34.	Meat, other	48.	Coriander
6.	*Suji* (cream of wheat/barley)	20.	Potato			49.	Cumin seeds
7.	Grains, other	21.	Radish	Food Category: Fish		50.	Dried chili
		22.	*Sheem* (bean)	35.	Catfish	51.	Dried turmeric
Food Category: Legumes		23.	Spinach	36.	Carp fish	52	Spices, other
8.	Anchor daal	24.	Water gourd	37.	Dried fish		
9.	Grass pea (*khesari*)	25.	Gourd, other	38.	Gura mach fish	Food Category: Miscellaneous	
10.	Lentil	26.	Leafy vegetables, other	39.	Panch mishali fish	53.	Biscuit
11.	Pulses and legumes, other			40.	Puti fish	54.	*Bonroti*/*paoroti*
		Food Category: Oils & Fats		41.	Rui fish	55.	Chips
Food Category: Vegetables		27.	Soyabean oil	42.	Taki fish	56.	Sugar
12.	Amaranth leaves	28.	Mustard oil	43.	Telapia fish	57.	Sweeteners, other
13.	*Dhania shak* (leafy vegetable)	29.	Oils and ghees, other	44.	Small fish, other	58.	Sweets, prepared
14.	Cauliflower			45.	Large fish, other	59.	Coconut

The list of fifty-nine foods is drawn from the original list of ninety-four food items, effectively showing that we are still able to capture 90 % of key nutrient intake and explain up to 90 % of between-person variability while also reducing the food list by thirty-five items. These thirty-five items that were excluded can be categorized primarily as spices, prepared foods and fruit subgroupings in the original ninety-four-item food list. On the other hand, the final food list (Table 5) represents a majority of the foods from the ninety-four-item list for the following food groups: grains, legumes, vegetables, oils and fats, fish and meat.

## Discussion

The objective of the current analysis was to demonstrate how the application of an epidemiological approach typically used for developing FFQ could be adapted and applied to develop a more comprehensive food list for an HCES consumption module in order to gather data relevant for household-level food and nutrient analyses. The final food list contains nine food groups and fifty-nine items which together account for 90 % of total intake and 90 % of between-person variance for energy and the five nutritional components analysed. If additional nutrients had been considered in the analysis, the food list would have had additional items, although likely many of these are accounted for by these foods (e.g. thiamin, riboflavin, niacin, folate).

The analysis uses 24 h dietary recall data from a large survey in rural Bangladesh to demonstrate how a nutrition-relevant food list can be created. However, for this food list to be fully operational for use within a typical HCES, several additional steps need to be carried out. First, care should be taken to ensure that the food list is compatible with the primary objectives of the HCES (e.g. to measure poverty). To address the first point, designers of HCES food lists should combine the food list derived from the traditional approach taken for an expenditure-based food list and compare the items with the food list derived based on nutrition-related interest in key foods and nutrients. To this list any additional food items should be added that are of particular policy relevance (e.g. potential foods for fortification). Any overlapping items should be removed, and a complete food list accounting for items of interest from an expenditure and a nutrition perspective should be included. Second, pre-testing of the food list is needed. Focus groups or cognitive interviews should be used to ensure that the food items appear in a clear and understandable way for enumerators and respondents alike, and that the grouping of the items is logical^(^ ^9^ ^,^ ^11^ ^)^. Special attention should be given to the way the items are represented to ensure clarity. In addition, the ordering of food items and food groups should be carefully considered to ensure that items are clustered in a way that makes sense to respondents and fits within their own cognitive framework. For example, one recent study identified during the cognitive interview phase that changing the order of certain items, such as placing plain porridge in front of flavoured porridge, reduced double counting^(^ ^11^ ^)^. Given the objective and nature of the present study, these steps were not conducted but would be required as part of the operationalization of the final food list.

There are several important factors that will determine how extensive the final food list is, including the season in which the 24 h dietary recall data were collected and the breadth of the nutrients of interest selected by the researcher at the outset of the analysis. In the current analysis each of these factors played an important role in the final food list. Of primary concern is the extent to which the 24 h dietary recall captures all foods in the diet throughout the year. If the 24 h dietary recall covers only one season, then certain foods may or may not be present, especially for foods that may only be consumed when they are in season (e.g. mangos). In the present study, data for the 24 h dietary recall were collected between October and March, which corresponds to the late autumn (*hemanta*) and winter (*shit*) in Bangladesh. As noted, the reported frequency of fruit consumption was very low, for example for bananas (3·25 %), apples (1·88 %), papayas (0·18 %) and mangos (0·03 %).

Another reason why some foods might not appear in the final food list relates to the selection of nutrients of interest, which should be determined by the researcher at the outset. For example, even though bananas were consumed by more than 2 % of the population, they did not end up in the food list possibly due to the restricted subset of nutrients that were considered for the analysis and exclusion of K as a nutrient of interest. A similar issue arises with citrus fruits not appearing on the food list despite being aggregated, likely due to the fact that vitamin C was not included as a nutrient of interest. There are several ways to remedy this situation. If the objective is to have a comprehensive food list, then all nutrients of public health significance should be included in the analysis and the 24 h dietary recall data should cover all seasons, with repeated samples from a subgroup of the study population to capture the usual diet throughout the year. If this option is not possible and the researchers are concerned that key items of public health interest are missing from the food list, these additional food items can be added to the food list at the discretion of the researchers based on their knowledge of the seasonal dietary patterns and local diets^(^ ^11^ ^)^. In the present case, for example, expanding the food list to include more fruits (e.g. mangos, papayas, bananas, apples, etc.) could permit analysis of the consumption of these foods and nutrients, particularly relevant if the HCES was being conducted during seasons in which these foods would be consumed. If there is concern about the list becoming too long, one possible solution, recommended by Cade *et al.* ^(^ ^9^ ^)^, is to include an additional section within the food list that asks specifically about foods that are more heavily consumed during certain seasons, which ensures that additional items are asked about only when appropriate based on a filter question or knowledge of the interviewer regarding seasonal consumption^(^ ^9^ ^)^.

The food items in the final food list should strike a balance between the specificity of individual food items and aggregated food items given the need to match foods with their nutrient composition using an FCT. A review of the food list makes it clear that for some items it will be straightforward to find a precise match in the FCT due to their specificity (e.g. ‘amaranth leaves’), while for other items it will be more difficult (e.g. ‘pulses and legumes, other’), which would require constructing an average of various individual foods. The objective should be to ensure as complete and precise a match between the food list and the available FCT as possible. One suggestion that has been put forward by the FAO is to include FoodEx2 coding, developed by the European Food Safety Authority, in FCT and in corresponding surveys to ensure a higher level of consistency in matching foods to the correct nutrient compositions (http://www.fao.org/gift-individual-food-consumption/en/).

In addition to the specificity of the individual food items, complete coverage of all major food groups is important. For purposes related to compiling and calculating consumer price indices, the UN Statistics Division has developed the Classification of Internal Consumption According to Purpose (COICOP)^(^ ^24^ ^)^. The COICOP is considered the international standard for classifying household expenditures (both food and non-food) in HCES. There are ten COICOP umbrella food groups, which are then broken down into more detailed sub-food groups and eventually individual food items. The COICOP umbrella food groups include: (i) cereals and cereal products; (ii) live land animals; (iii) fish and other seafood; (iv) milk, dairy products and eggs; (v) oils and fats; (vi) fruits and nuts; (vii) vegetables, tubers, plaintains and pulses; (viii) sugar, confectionery and dessert; (ix) ready-made food and other products; and (x) non-alcoholic beverages^(^ ^25^ ^)^. While some of these groupings are not conventional from a nutrition perspective (e.g. grouping together vegetables, tubers, plaintains and pulses), these food items can be grouped differently in the actual survey according to what makes intuitive sense to respondents and later re-grouped for analysis, for example to assess household dietary diversity. It is critical that food items are distinct and do not cover multiple categories. For example ‘canned fruits or vegetables’ should not be included as a single food item since this spans the Fruits and Vegetables groups^(^ ^2^ ^)^. Ultimately the final food list should contain sufficiently disaggregated food items from all food groups to ensure relevance for both expenditure and nutrition analyses.

Finally, an overarching consideration before conducting a similar analysis is that many low- and middle-income countries may not have a source of nationally representative 24 h dietary recall data that has been recently collected or is publicly available. When these data do exist, they provide a valuable resource, primarily for deriving accurate estimates of nutrient intakes of individuals disaggregated by age and sex groups and by anthropometric status, socio-economic status and education level, for example. However, if 24 h dietary recall data are available, they can allow for the development of a food list that can be used in between future 24 h dietary recall rounds, and will add great value to HCES by allowing for analyses that link agricultural production, socio-economic status and consumption. Therefore, household-level data should be viewed as a necessary complement, not a substitute, for individual-level dietary data.

### Other considerations and limitations

Some considerations and limitations of the present analysis have already been discussed, such as the lack of composite dishes, the grouping and ordering of items in the food list, and seasonality. Several others are mentioned here briefly for completeness. The underlying data for the current analysis are representative of rural Bangladesh and thus the food list does not take into account foods that are likely consumed in higher frequency in urban Bangladesh, such as prepared composite foods consumed outside the home and processed foods. This would need to be taken into consideration when comparing the nutrition-relevant food list derived from 24 h dietary data from rural Bangladesh with a food list from a national HCES, as it could affect the comparison. Furthermore, the diet in general in Bangladesh relies heavily on the staple crop of rice, which is a key source of energy, protein, Fe and Zn. In a country with a more varied diet and less reliance on a single food, more foods might be required to reach the 90 % threshold for contribution and explanation of variance.

In addition, the 24 h dietary recall survey was only administered once (no repeat survey to evaluate usual intake) and a single person (the food preparer) reported all food consumption on behalf of the entire household. Depending on the respondents’ awareness of the intake of all household members and the extent to which individuals consume food outside of home, relying on the BIHS method of capturing food intake could result in inaccurate estimation of consumption and probable underestimation of processed foods and composite dishes that would be more likely to be consumed outside the home.

Another limitation is the food grouping process. For this step, we followed examples in existing literature: we grouped conceptually similar foods that were consumed by a small proportion of the population, maintaining the unique identity of foods whenever they were consumed by 2 % or more of the population. The main issue is that this approach could be difficult to replicate, since some discretion is left to the analyst to determine which foods should be grouped together and which cut-offs should be applied. Several different approaches have been used in the literature. For example, foods are grouped based on similarity of food category, nutrient content and limited consumption of the food^(^ ^20^ ^)^; based on form, nutrient density and type of preparation method^(^ ^21^ ^)^; or by combining conceptually similar foods based on fat and energy per portion eaten for most foods, except for fruit and vegetables, where aggregations are based on vitamin and mineral content^(^ ^22^ ^)^. Given the importance of this step, both an analyst and a nutritionist should be available to look over the final aggregations of food items and ensure that they are appropriately grouped based on the predetermined parameters. This serves as a reminder that getting the food list ‘right’ for HCES from a food and nutrition perspective requires careful consideration to ensure that the food list serves its traditional purpose to capture the key foods of household expenditure for poverty analyses, while also encompassing a broader range of foods that contribute to the bulk of the population’s dietary patterns and nutrient intake.

## Conclusion

Due to the heterogeneity of surveys across countries, it is difficult to make overarching statements about HCES, but in many instances the food lists lack specificity and may not be representative of the national diet. For researchers and decision makers who wish to add household-level food and nutrition analyses to the common purposes of the HCES, these shortcomings pose a challenge as they may make it difficult to match foods with their nutrient profiles in the FCT and may exclude some nutritionally significant foods, resulting in poor consumption estimates for use in food and nutrition applications.

The present paper demonstrates that following a simple and systematic method derived from nutritional epidemiology can contribute to the development of a robust food list for use in an HCES that can be used to capture information on nutrient intake across a range of key nutrients. The analysis showed that using a data-driven approach and combining rankings based on both total intake and between-person variation can result in a parsimonious and comprehensive food list for rural Bangladesh that captures 90 % of total nutrient intake and 90 % of between-person variance in the consumption of key nutrients. This is a critical first step in the process of operationalizing a list-based recall for use in an HCES. To ensure that the food list is aligned with the traditional needs of the HCES and to make sure that the food list is clear, additional steps are necessary, such as ensuring that all key expenditure food items are in the list; pre-testing the food list with enumerators and respondents; ensuring the food groups and ordering of foods makes sense; and estimating the amounts of foods consumed. In addition, there are several non-trivial matters that need to be addressed, including identifying an existing 24 h dietary recall; determining which dietary components are of interest; achieving precision of the food matches during the nutrient analytic phase when matching with the FCT; constructing appropriate food groupings; and assessing the seasonal coverage and quality of the underlying 24 h dietary recall. Consideration must be given to potential trade-offs with other objectives of a typical HCES consumption module and how best to meet the full range of objectives. The method proposed in the present paper is a first step in resolving many of these challenges.

## Footnotes

* There are 292 items in the original food list; however, our list was based on 288 items because we removed three non-food items (betel nut, betel leaf, tobacco) and a duplicate food in the food list, which we collapsed into a single food, ultimately resulting in a list of 288 foods.

## Supplementary material

For supplementary material accompanying this paper visit https://doi.org/10.1017/S1368980018002847. click here to view supplementary material
