# Supplementary material for: Getting the food list ‘right’: an approach for the development of nutrition-relevant food lists for household consumption and expenditure surveys
Source: Public Health Nutr. 2018 Nov 5;22(2):246–56. doi: 10.1017/S1368980018002847 (PMC6390398; doi:10.1017/S1368980018002847)
Supplement: Supplementary file 1 [file S1368980018002847sup001.docx]

| *Table: Top twenty food contributors to total protein intake (n=94)* | | | | |
| --- | --- | --- | --- | --- |
|  |  | Contribution to total intake | | |
| Rank | Food item | Percentage | Cumulative percentage | Percentage of population that consumes food |
| 1 | Rice, coarse | 48.59 | 48.59 | 89.52 |
| 2 | Rice, fine | 5.64 | 54.24 | 12.96 |
| 3 | Atta (wheat flour) | 3.62 | 57.86 | 15.09 |
| 4 | Dried fish | 3.14 | 61.00 | 14.34 |
| 5 | Large fish, other | 3.03 | 64.03 | 16.57 |
| 6 | Lentil | 2.45 | 66.48 | 13.32 |
| 7 | Chicken | 2.14 | 68.61 | 6.31 |
| 8 | Beef/buffalo | 2.12 | 70.74 | 4.77 |
| 9 | Small fish, other | 2.11 | 72.84 | 11.08 |
| 10 | Potato | 1.78 | 74.62 | 80.88 |
| 11 | Carp fish | 1.57 | 76.19 | 8.55 |
| 12 | Pangash/catfish | 1.08 | 77.27 | 6.21 |
| 13 | Egg | 1.04 | 78.31 | 15.42 |
| 14 | Puti fish | 1.01 | 79.32 | 6.06 |
| 15 | Rui fish | 1.00 | 80.32 | 6.09 |
| 16 | Eggplant | 0.99 | 81.31 | 31.60 |
| 17 | Panch mishali fish | 0.98 | 82.29 | 4.94 |
| 18 | Sheem (bean) | 0.97 | 83.26 | 27.57 |
| 19 | Milk | 0.89 | 84.15 | 18.15 |
| 20 | Muri/khoi (puffed rice) | 0.84 | 84.99 | 16.88 |

| *Table: Top twenty food contributors to total fat intake (n=94)* | | | | |
| --- | --- | --- | --- | --- |
|  |  | Contribution to total intake | | |
| Rank | Food item | Percentage | Cumulative percentage | Percentage of population that consumes food |
| 1 | Soybean oil | 53.24 | 53.24 | 83.02 |
| 2 | Mustard oil | 9.92 | 63.16 | 31.47 |
| 3 | Rice, coarse | 9.85 | 73.01 | 89.52 |
| 4 | Coconut | 2.69 | 75.70 | 2.76 |
| 5 | Large fish, other | 2.05 | 77.75 | 16.57 |
| 6 | Egg | 1.49 | 79.24 | 15.42 |
| 7 | Biscuit | 1.32 | 80.56 | 26.81 |
| 8 | Atta (wheat flour) | 1.23 | 81.79 | 15.09 |
| 9 | Beef/buffalo | 1.20 | 82.99 | 4.77 |
| 10 | Milk | 1.09 | 84.08 | 18.15 |
| 11 | Dried fish | 0.98 | 85.06 | 14.34 |
| 12 | Small fish, other | 0.97 | 86.03 | 11.08 |
| 13 | Carp fish | 0.96 | 86.99 | 8.55 |
| 14 | Chips | 0.78 | 87.77 | 8.11 |
| 15 | Sweets, prepared | 0.77 | 88.55 | 11.75 |
| 16 | Chicken | 0.75 | 89.30 | 6.31 |
| 17 | Turmeric (dried) | 0.59 | 89.89 | 95.26 |
| 18 | Puti fish | 0.57 | 90.45 | 6.06 |
| 19 | Fried food | 0.53 | 90.98 | 5.27 |
| 20 | Dried chili | 0.52 | 91.50 | 69.19 |

| *Table: Top twenty food contributors to total iron intake (n=94)* | | | | |
| --- | --- | --- | --- | --- |
|  |  | Contribution to total intake | | |
| Rank | Food item | Percentage | Cumulative percentage | Percentage of population that consumes food |
| 1 | Rice, coarse | 32.89 | 32.89 | 89.52 |
| 2 | Atta (wheat flour) | 7.37 | 40.27 | 15.09 |
| 3 | Turmeric (dried) | 6.02 | 46.28 | 95.26 |
| 4 | Rice, fine | 3.88 | 50.16 | 12.96 |
| 5 | Potato | 3.80 | 53.96 | 80.88 |
| 6 | Leafy vegetables, other | 2.95 | 56.92 | 9.81 |
| 7 | Dried fish | 2.74 | 59.66 | 14.34 |
| 8 | Amaranth leaves | 2.74 | 62.41 | 6.92 |
| 9 | Jira (cumin seeds) | 2.69 | 65.09 | 49.55 |
| 10 | Lentil | 2.27 | 67.36 | 13.32 |
| 11 | Sheem (bean) | 1.85 | 69.22 | 27.57 |
| 12 | Onion | 1.68 | 70.89 | 95.95 |
| 13 | Water gourd | 1.52 | 72.41 | 7.70 |
| 14 | Anchor daal | 1.29 | 73.70 | 4.27 |
| 15 | Small fish, other | 1.25 | 74.95 | 11.08 |
| 16 | Lau shak (bottle gourd leaves) | 1.11 | 76.05 | 5.19 |
| 17 | Beef/buffalo | 1.09 | 77.14 | 4.77 |
| 18 | Large fish, other | 1.05 | 78.20 | 16.57 |
| 19 | Suji (cream of wheat/barley) | 0.99 | 79.19 | 3.50 |
| 20 | Eggplant | 0.96 | 80.15 | 31.60 |

| *Table: Top twenty food contributors to total zinc intake (n=94)* | | | | |
| --- | --- | --- | --- | --- |
|  |  | Contribution to total intake | | |
| Rank | Food item | Percentage | Cumulative percentage | Percentage of population that consumes food |
| 1 | Rice, coarse | 49.68 | 49.68 | 89.52 |
| 2 | Potato | 7.39 | 57.07 | 80.88 |
| 3 | Rice, fine | 5.86 | 62.93 | 12.96 |
| 4 | Atta (wheat flour) | 4.76 | 67.69 | 15.09 |
| 5 | Beef/buffalo | 2.52 | 70.21 | 4.77 |
| 6 | Lentil | 2.16 | 72.37 | 13.32 |
| 7 | Eggplant | 1.86 | 74.22 | 31.60 |
| 8 | Chicken | 1.23 | 75.45 | 6.31 |
| 9 | Green chili | 1.13 | 76.58 | 71.68 |
| 10 | Puti fish | 1.08 | 77.66 | 6.06 |
| 11 | Egg | 1.03 | 78.69 | 15.42 |
| 12 | Sheem (bean) | 0.94 | 79.63 | 27.57 |
| 13 | Large fish, other | 0.93 | 80.55 | 16.57 |
| 14 | Onion | 0.91 | 81.46 | 95.95 |
| 15 | Turmeric (dried) | 0.85 | 82.32 | 95.26 |
| 16 | Small fish, other | 0.83 | 83.14 | 11.08 |
| 17 | Milk | 0.82 | 83.97 | 18.15 |
| 18 | Gura mach (small fish) | 0.71 | 84.68 | 4.61 |
| 19 | Rice flour | 0.68 | 85.36 | 5.74 |
| 20 | Dried chili | 0.67 | 86.03 | 69.19 |

| *Table: Between-person variation and total protein intake for the top 15 foods (n=94)* | | | | | | | | |
| --- | --- | --- | --- | --- | --- | --- | --- | --- |
| Stage of entry into regression* |  | Households consuming specific food (%) |  | Contribution to between-person variation of protein |  | Contribution to total protein intake | | |
|  | Food item |  |  | Cumulative R^2^ |  | Rank for total intake** | Percentage | Cumulative percentage |
| 1 | Rice, coarse | 89.52 |  | 0.334 |  | 1 | 48.59 | 48.59 |
| 2 | Rice, fine | 12.96 |  | 0.573 |  | 2 | 5.64 | 54.24 |
| 3 | Beef/buffalo | 4.77 |  | 0.646 |  | 8 | 2.12 | 56.36 |
| 4 | Atta (wheat flour) | 15.09 |  | 0.702 |  | 3 | 3.62 | 59.98 |
| 5 | Chicken | 6.31 |  | 0.747 |  | 7 | 2.14 | 62.12 |
| 6 | Dried fish | 14.34 |  | 0.78 |  | 4 | 3.14 | 65.26 |
| 7 | Small fish, other | 11.08 |  | 0.808 |  | 9 | 2.11 | 67.37 |
| 8 | Lentil | 13.32 |  | 0.826 |  | 6 | 2.45 | 69.82 |
| 9 | Large fish, other | 16.57 |  | 0.843 |  | 5 | 3.03 | 72.84 |
| 10 | Rice flour | 5.74 |  | 0.859 |  | 21 | 0.81 | 73.65 |
| 11 | Birds, other | 0.85 |  | 0.87 |  | 37 | 0.37 | 74.01 |
| 12 | Onion | 95.95 |  | 0.879 |  | 30 | 0.49 | 74.50 |
| 13 | Meat, other | 0.86 |  | 0.887 |  | 44 | 0.28 | 74.78 |
| 14 | Carp fish | 8.55 |  | 0.894 |  | 11 | 1.57 | 76.35 |
| 15 | Rui fish | 6.09 |  | 0.901 |  | 15 | 1.00 | 77.35 |
| *Stage of entry based on contribution to variance in consumption, ranked high to low | | | | | | | |  |
| **Ranked in order of highest to lowest contributor to total nutrient | | | | | | |  |  |

| *Table: Between-person variation and total fat intake for the top 15 foods (n=94)* | | | | | | | | |
| --- | --- | --- | --- | --- | --- | --- | --- | --- |
| Stage of entry into regression* |  | Households consuming specific food (%) |  | Contribution to between-person variation of fat |  | Contribution to total fat intake | | |
|  | Food item |  |  | Cumulative R^2^ |  | Rank for total intake** | Percentage | Cumulative percentage |
| 1 | Soybean oil | 83.02 |  | 0.63 |  | 1 | 53.24 | 53.24 |
| 2 | Mustard oil | 31.47 |  | 0.795 |  | 2 | 9.92 | 63.16 |
| 3 | Coconut | 2.76 |  | 0.899 |  | 4 | 2.69 | 65.85 |
| 4 | Oils and ghees, other | 1.00 |  | 0.915 |  | 22 | 0.48 | 66.33 |
| 5 | Rice, coarse | 89.52 |  | 0.924 |  | 3 | 9.85 | 76.19 |
| 6 | Beef/buffalo | 4.77 |  | 0.933 |  | 9 | 1.20 | 77.39 |
| 7 | Large fish, other | 16.57 |  | 0.942 |  | 5 | 2.05 | 79.43 |
| 8 | Sweets, prepared | 11.75 |  | 0.948 |  | 15 | 0.77 | 80.21 |
| 9 | Meat, other | 0.86 |  | 0.953 |  | 29 | 0.31 | 80.52 |
| 10 | Biscuit | 26.81 |  | 0.958 |  | 7 | 1.32 | 81.84 |
| 11 | Egg | 15.42 |  | 0.962 |  | 6 | 1.49 | 83.33 |
| 12 | Fried food | 5.27 |  | 0.965 |  | 19 | 0.53 | 83.86 |
| 13 | Milk | 18.15 |  | 0.968 |  | 10 | 1.09 | 84.95 |
| 14 | Chips | 8.11 |  | 0.971 |  | 14 | 0.78 | 85.74 |
| 15 | Atta (wheat flour) | 15.09 |  | 0.974 |  | 8 | 1.23 | 86.96 |
| *Stage of entry based on contribution to variance in consumption, ranked high to low | | | | | | |  |  |
| **Ranked in order of highest to lowest contributor to total nutrient | | | | |  |  |  |  |

| *Table: Between-person variation and total fat intake for the top 15 foods (n=94)* | | | | | | | | |
| --- | --- | --- | --- | --- | --- | --- | --- | --- |
| Stage of entry into regression* |  | Households consuming specific food (%) |  | Contribution to between-person variation of iron |  | Contribution to total iron intake | | |
|  | Food item |  |  | Cumulative R^2^ |  | Rank for total intake** | Percentage | Cumulative percentage |
| 1 | Rice, coarse | 89.52 |  | 0.203 |  | 1 | 32.89 | 32.89 |
| 2 | Atta (wheat flour) | 15.09 |  | 0.376 |  | 2 | 7.37 | 40.27 |
| 3 | Rice, fine | 12.96 |  | 0.539 |  | 4 | 3.88 | 44.14 |
| 4 | Turmeric (dried) | 95.26 |  | 0.604 |  | 3 | 6.02 | 50.16 |
| 5 | Amaranth leaves | 6.92 |  | 0.649 |  | 8 | 2.74 | 52.90 |
| 6 | Grains, other | 3.48 |  | 0.683 |  | 23 | 0.87 | 53.78 |
| 7 | Soybean oil | 83.02 |  | 0.712 |  | 75 | 0.07 | 53.85 |
| 8 | Leafy vegetables, other | 9.81 |  | 0.739 |  | 6 | 2.95 | 56.80 |
| 9 | Dried fish | 14.34 |  | 0.761 |  | 7 | 2.74 | 59.55 |
| 10 | Suji (cream of wheat/barley) | 3.50 |  | 0.784 |  | 19 | 0.99 | 60.54 |
| 11 | Jira (cumin seeds) | 49.55 |  | 0.806 |  | 9 | 2.69 | 63.23 |
| 12 | Spices, other | 2.49 |  | 0.824 |  | 49 | 0.31 | 63.54 |
| 13 | Anchor daal | 4.27 |  | 0.84 |  | 14 | 1.29 | 64.83 |
| 14 | Small fish, other | 11.08 |  | 0.856 |  | 15 | 1.25 | 66.07 |
| 15 | Beef/buffalo | 4.77 |  | 0.869 |  | 17 | 1.09 | 67.16 |
| *Stage of entry based on contribution to variance in consumption, ranked high to low | | | | | | |  |  |
| **Ranked in order of highest to lowest contributor to total nutrient | | | | |  |  |  |  |

| *Table: Between-person variation and total iron intake for the top 15 foods (n=94)* | | | | | | | | |
| --- | --- | --- | --- | --- | --- | --- | --- | --- |
| Stage of entry into regression* |  | Households consuming specific food (%) |  | Contribution to between-person variation of zinc |  | Contribution to total zinc intake | | |
|  | Food item |  |  | Cumulative R^2^ |  | Rank for total intake** | Percentage | Cumulative percentage |
| 1 | Rice, coarse | 89.52 |  | 0.345 |  | 1 | 49.68 | 49.68 |
| 2 | Rice, fine | 12.96 |  | 0.591 |  | 3 | 5.86 | 55.53 |
| 3 | Atta (wheat flour) | 15.09 |  | 0.695 |  | 4 | 4.76 | 60.30 |
| 4 | Beef/buffalo | 4.77 |  | 0.812 |  | 5 | 2.52 | 62.81 |
| 5 | Soybean oil | 83.02 |  | 0.836 |  | 80 | 0.02 | 62.83 |
| 6 | Meat, other | 0.86 |  | 0.853 |  | 37 | 0.35 | 63.18 |
| 7 | Chicken | 6.31 |  | 0.865 |  | 8 | 1.23 | 64.41 |
| 8 | Lentil | 13.32 |  | 0.876 |  | 6 | 2.16 | 66.57 |
| 9 | Puti fish | 6.06 |  | 0.886 |  | 10 | 1.08 | 67.64 |
| 10 | Potato | 80.88 |  | 0.895 |  | 2 | 7.39 | 75.04 |
| 11 | Rice flour | 5.74 |  | 0.904 |  | 19 | 0.68 | 75.72 |
| 12 | Muri/khoi (puffed rice) | 16.88 |  | 0.91 |  | 23 | 0.65 | 76.37 |
| 13 | Small fish, other | 11.08 |  | 0.916 |  | 16 | 0.83 | 77.20 |
| 14 | Pulses and legumes, other | 4.06 |  | 0.921 |  | 27 | 0.53 | 77.73 |
| 15 | Gura mach (small fish) | 4.61 |  | 0.926 |  | 18 | 0.71 | 78.43 |
| *Stage of entry based on contribution to variance in consumption, ranked high to low | | | | | | | |  |
| **Ranked in order of highest to lowest contributor to total nutrient | | | | | |  |  |  |
